# Brief report: RRx-001 is a c-Myc inhibitor that targets cancer stem cells

**DOI:** 10.18632/oncotarget.25211

**Published:** 2018-05-04

**Authors:** Bryan Oronsky, Tony R. Reid, Arnold Oronsky, Scott Caroen, Corey A. Carter, Pedro Cabrales

**Affiliations:** ^1^ EpicentRx Inc, San Diego, CA 92121, USA; ^2^ Moores Cancer Center, University of California San Diego, La Jolla, CA 92093, USA; ^3^ InterWest Partners, Menlo Park, CA 94025, USA; ^4^ Department of Bioengineering, University of California San Diego, La Jolla, CA 92093, USA

**Keywords:** cancer stem cell, c-Myc, Wnt pathway, RRx-001, resistance reversal

## Abstract

The goal of anticancer therapy is to selectively eradicate all malignant cells. Unfortunately for the majority of patients with metastatic disease, this goal is consistently thwarted by the nearly inevitable development of therapeutic resistance; the main driver of therapeutic resistance is a minority subpopulation of cancer cells called cancer stem cells (CSCs) whose mitotic quiescence essentially renders them non-eradicable. The Wnt signaling pathway has been widely implicated as a regulator of CSCs and, therefore, its inhibition is thought to result in a reversal of therapeutic resistance via loss of stem cell properties.

RRx-001 is a minimally toxic redox-active epi-immunotherapeutic anticancer agent in Phase III clinical trials that sensitizes tumors to radiation and cytotoxic chemotherapies. In this article, as a potential mechanism for its radio- and chemosensitizing activity, we report that RRx-001 targets CD133**^+^**/CD44**^+^** cancer stem cells from three colon cancer cell-lines, HT-29, Caco-2, and HCT116, and inhibits Wnt pathway signalling with downregulation of c-Myc.

## INTRODUCTION

The c-Myc (Myc) oncoprotein is a well-established driver of breast, lung, colorectal and prostate cancers that is currently considered “undruggable” [1–4]. Myc overexpression is associated with cancer stem cell maintenance [5] and, by extension, with therapeutic resistance since cancer stem cells are thought to be the primary mediators of tumor resistance and progression. RRx-001 is a first-in-class minimally toxic epi-immunotherapeutic agent in Phase III clinical trials as a chemosensitizer to reverse resistance in small cell lung cancer (SCLC), high-grade neuroendocrine carcinomas (HGNEC) and colorectal cancers [6].

Herein, as a potential mechanism for its radio- and chemosensitizing activity, we demonstrate for the first time that RRx-001 targets cancer stem cells (CSCs) and that it decreases the expression levels of Wnt pathway components and target genes, such as TCF4, Pygo2, Axin2 and c-Myc, which are known to govern stem cell renewal and differentiation [7, 8].

## RESULTS

### RRx-001 selectively targets colon CSCs

CD133 and CD44 are widely considered as markers of cancer stem/progenitor-like cells. In order to determine the effect of RRx-001 on both cancer and CSC cell proliferation, an MTT assay was performed.

**Figure 1:**
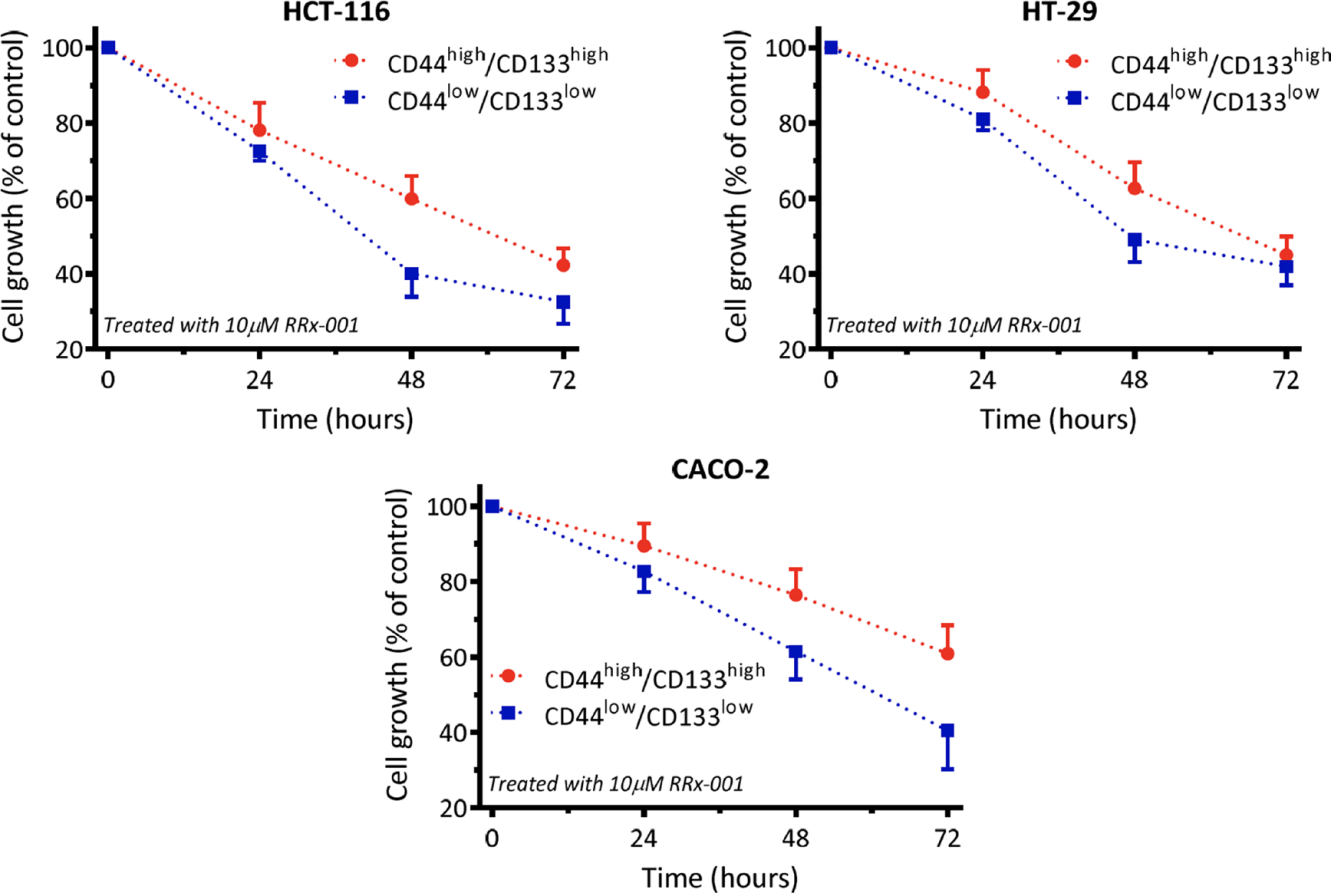
RRx-001 inhibits proliferation of colon cancer cells and stem cells in all three cell lines

Results of the flow cytometric assay depicted that after 3-day treatment, RRx-001 reduced the CD133^+^/CD44^+^ population in a time-dependent manner in all three cell lines. The extent of reduction was less than that of non-CSC cancer cells (Figure 1).

### RRx-001 downregulates components of the Wnt pathway

**Figure 2:**
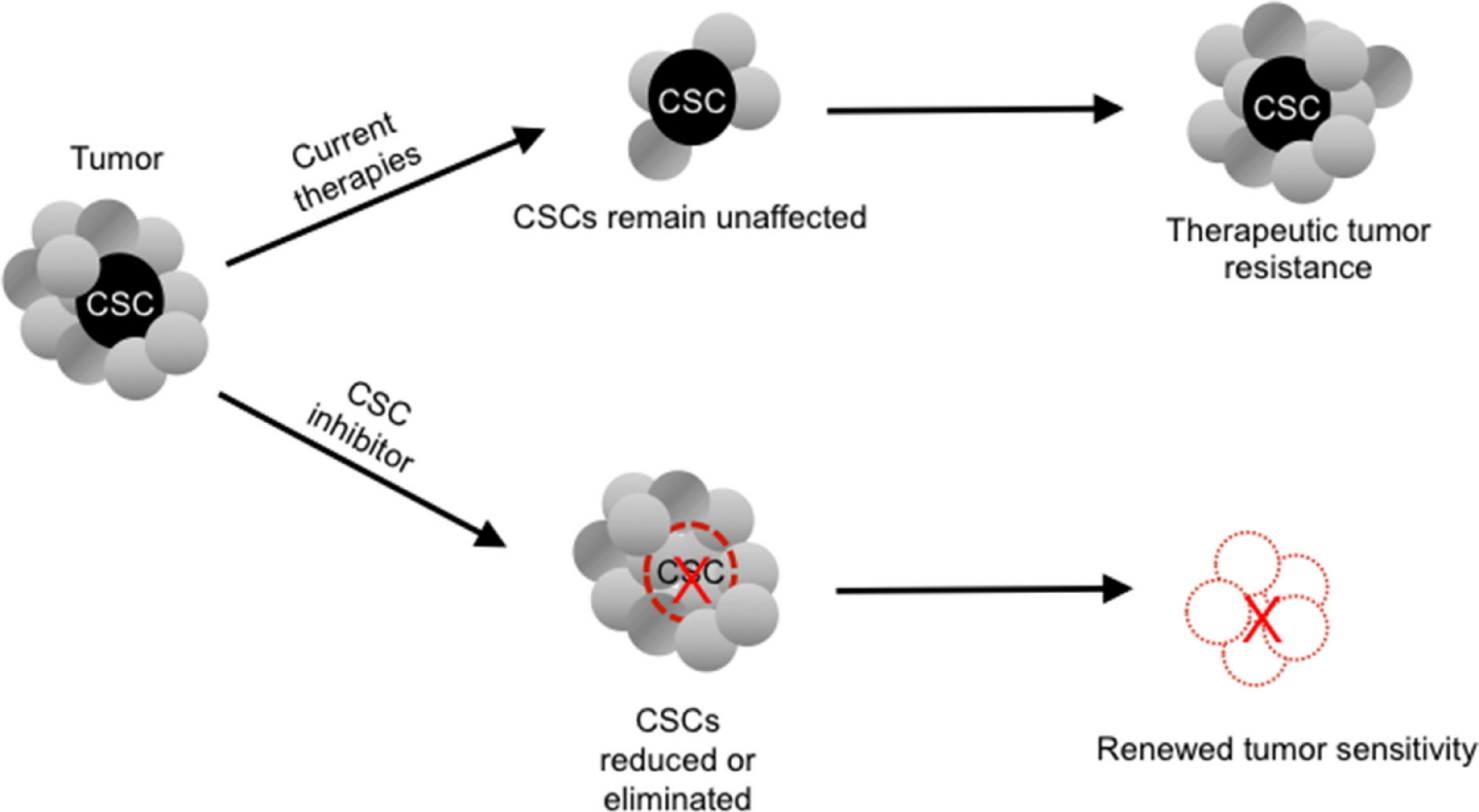
Potential paradigm-shifting effect of a cancer stem cell (CSC) inhibitor on tumor chemoresistance

**Figure 3:**
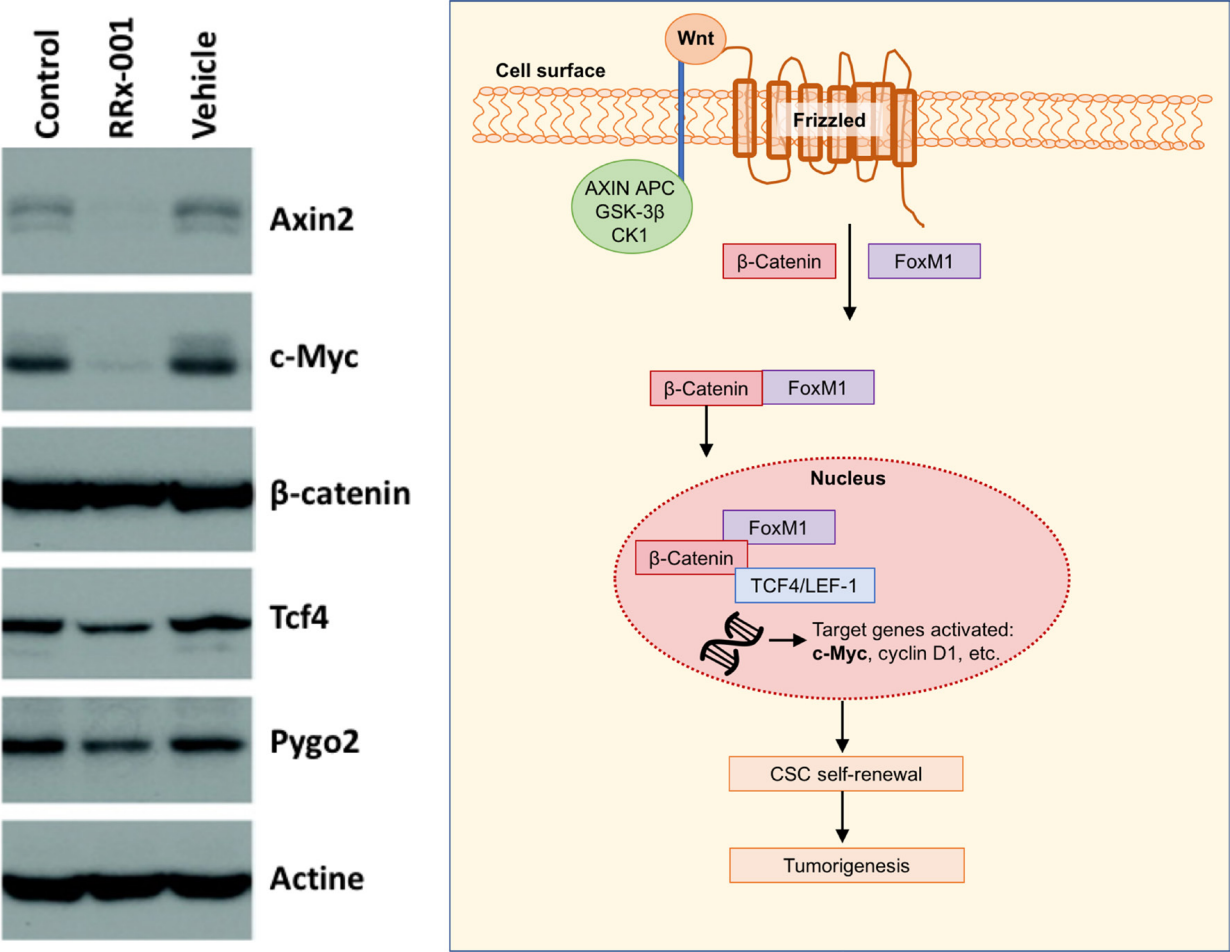
Western blot of analysis of Wnt target genes with Wnt pathway shown to the right

To test the hypothesis that downregulation of the Wnt signalling pathway is responsible for the reduced CSC growth, since the Wnt signaling pathway is reported to sustain self-renewal potential and chemoresistance in CSCs [9, 10], the impact of RRx-001 on the Wnt pathway was investigated. (Figure 2) As shown in Figure 3, RRx-001 dramatically decreased expression levels of Axin2 and c-Myc.

## DISCUSSION

In light of the failure of currently available therapeutic options to eliminate CSCs, which are associated with treatment resistance and cancer recurrence, CSC-targeted therapies have the potential to usher in a new paradigm shift in oncology (Figure 3).

## MATERIALS AND METHODS

### RRx-001

RRx-001, provided by EpicentRx, Inc, was diluted in dimethylsulfoxide (DMSO) to a 10 μM concentration.

### Fluorescence-activated cell sorting (FACS) of cells

Colon cancer cell lines Caco-2, HT-29, and HCT-116 were obtained from the American Type Culture Collection (ATCC, Rockville, MD, USA), and maintained according to the ATCC's instructions. All culture reagents were from Invitrogen (Carlsbad, CA, USA). To analyze the expression of CD44 and CD133 in different lines, cells were treated with 10 μM RRx-001 for 72 hours, then trypsin-digested and re-suspended in stain buffer (1 × 10^6^ cells in 80 μl). Cells were then treated with 20 μl FcR Blocking Reagent for 15 min, and incubated with antibodies (human anti-CD44-FITC and human anti-CD133-PE) for 30 min. After staining, cells were subjected to flow cytometry.

To isolate CD44^high^/CD133^high^ and CD44^low^/CD133^low^ cells were trypsinized and blocked with FcR Blocking Reagent. Propidium iodide staining was applied to exclude the dead cells. Live cells were incubated with antibodies (human anti-CD44-FITC and human anti-CD133-APC) for 30 min. CD44^low^/CD133^low^ and CD44^high^/CD133^high^ cells were sorted by a cell sorter.

### Cell proliferation measurement

The colon cancer cells were split into 96 well dishes at 2,000 cells per well. Cells were treated with 10 μM of RRx-001. Cell proliferation was evaluated using the MTT (3-[4,5-dimethylthiazol-2-yl]-2,5-diphenyl tetrazolium bromide) assay at indicated days. The absorbance was measured at the wavelength of 570 nm. The measured optical density (OD) values were directly proportional to the number of viable cells. Then,

dose-response curves were fitted to the data. All experiments were repeated at least three times.

### Western blot for protein expression

Whole-cell lysates were separated by 10% SDS–PAGE and proteins were analyzed by western blotting for the expression of Axin 2, c-Myc, β-catenin, Tcf4, and Pygo2. Actin was used as a loading control.

### Statistical analysis

All data were presented as mean ± SD from three sets of independent experiments and analyzed using statistical software GraphPad Prism. Statistical differences between groups were determined by unpaired Student's *t*-test or One-way Analysis of Variance (ANOVA); *P* < 0.05 was considered as statistical significance.

## CONCLUSIONS

Herein, we demonstrate for the first time that RRx-001 targets colon CSCs and inhibits multiple components of the self-renewal Wnt pathway including c-Myc. Since Myc is not an easily “druggable” protein, due to a lack of enzymatic activity or any small molecule-binding deep pockets [11], these results suggest that RRx-001 should be evaluated as a treatment for c-Myc-overexpressing tumors.
